# Patient safety ¿ perspectives on evidence, information, and knowledge transfer

**DOI:** 10.1186/s13037-014-0033-6

**Published:** 2014-08-23

**Authors:** Philip F Stahel

**Affiliations:** 1 Department of Orthopaedics, Denver Health Medical Center, University of Colorado Denver, School of Medicine, 777 Bannock Street, Denver 80204, CO, USA

## Abstract

No abstract.

## 

The published literature on patient safety principles is largely anecdotal and the evidence-based knowledge in the field remains scarce. Lorri Zipperer, the editor of this new and groundbreaking book (Box), has to be commended for her effort in providing a fresh perspective on the impact of ¿EI&K¿ (*Evidence, Information, Knowlegde*) principles for patient safety. The new book appears to successfully complete the daunting task of extrapolating the ¿dry¿ theoretical aspects of interest to librarians and patient safety professionals, to relevant clinical processes and applications of interest to practicing physicians (including surgeons). 

The foreword by Robert M. Wachter, MD, a globally respected expert in the field of medical errors and patient safety, provides weight and credibility to the authoritarian nature of this new work.

The textbook is structured into five main sections. These include an introductory part on the basic context for innovation and improvement, followed by three specific sections devoted to the ¿EI&K¿ paradigm and its practical applicability in patient safety, and finalized by a section dedicated to future perspectives. Each chapter is written by distinguished experts in the field. Multiple tables, ¿infoboxes¿, and algorithms enhance the readibility of the individual chapters. In addition, the appendices at the end of the book serve as a valued resource to the reader. For example, appendix 1 provides a concise and highly appealing historic timeline on the evolution of patient safety, stratified from a ¿sporadic period¿ and a ¿cult period¿ to the current ¿breakout period¿ of the 21st century following the publication of the Institute of Medicine¿s seminal report, *To Err is Human*, in 1999. The standardized structure of providing boxes with specific case examples, as well as relevant take-home messages at the end of each chapter (¿key take-aways¿), allows for the reader to easily comprehend the complex content of this book, particularly from a clinician¿s perspective.

The key objective of this textbook is to inform healthcare professionals about the relevance of EI&K to core principles of patient safety, and their applicability in the clinical setting. The book furthermore raises awareness of the potential for failure of EI&K processes in the extrapolation to the clinical practice, and outlines the underlying root causes of failure.

Beyond a doubt, this meritorious work was desperately needed, and will hopefully allow to foster the ¿buy-in¿ and support by healthcare providers in sustaining and growing the current global momentum of the patient safety initiative.

